# PINK1: From Parkinson’s disease to mitophagy and back again

**DOI:** 10.1371/journal.pbio.3002196

**Published:** 2023-06-29

**Authors:** Benjamin O’Callaghan, John Hardy, Helene Plun-Favreau

**Affiliations:** 1 Department of Neurodegenerative Disease, UCL Queen Square Institute of Neurology, London, United Kingdom; 2 Aligning Science Across Parkinson’s (ASAP) Collaborative Research Network, Chevy Chase, Maryland, United States of America; 3 UCL Dementia Research Institute, London, United Kingdom

## Abstract

The genetics of Parkinson’s disease has been key to unravelling the PINK1-dependent mitophagy process. Here, we discuss the implications of a 2010 *PLOS Biology* paper that shed light on the functional importance of PINK1 in the mitophagy cascade.

## Abstract 2

PINK1-dependent mitophagy is thought to be a factor in Parkinson’s disease and is being investigated for therapeutic targeting. This Perspective looks back at a 2010 PLOS Biology paper that shed light on the functional importance of PINK1 in the mitophagy cascade.

This article is part of the *PLOS Biology* 20th Anniversary Collection.

The identification of pathogenic genetic variants can provide great insight into underlying disease mechanisms and reveal novel avenues for therapeutic intervention. Genetic perturbations that cause pathologically overlapping clinical phenotypes are often involved in shared cellular processes. The association of novel genes with a particular disease can therefore facilitate identification and deeper mechanistic understanding of human biological pathways. An illustration of this is the discovery of the PINK1–Parkin-dependent mitophagy pathway through initial genetic associations with familial early-onset recessive Parkinson’s disease (PD).

The E3 ubiquitin ligase Parkin was the first piece added to the mitophagy puzzle, with early work in human cell lines showing Parkin is trafficked from the cytoplasm to depolarized or damaged mitochondria, where it mediates the subsequent recruitment of autophagy components for mitochondrial degradation through the autophagy–lysosomal pathway [1,2]. At first, it was unclear how Parkin distinguishes depolarized mitochondria from those which are healthy. In a landmark paper published in 2010 in *PLOS Biology* [3], Narendra and colleagues greatly contributed to unravelling the requisite functional importance of the mitochondrial kinase PINK1 within this mitophagy cascade (Fig 1A). In agreement with other articles from the time, they showed that while in basal conditions, endogenous levels of PINK1 protein are generally low and undetectable within cells in culture, PINK1 accumulates at the surface of mitochondria following their depolarization. PINK1 recruitment was unaffected by the loss of Parkin, yet the converse knockout of PINK1 prevented both the recruitment of Parkin and downstream mitochondrial degradation through autophagy, providing a mechanistic explanation for previous observations in *Drosophila* that suggested PINK1 acted upstream of Parkin. It was already known that PINK1 is cleaved in a mitochondrial membrane potential–dependent manner and that the cleavage products are vulnerable to rapid degradation via the proteosome. But it was Narendra and colleagues who first implicated this process in selective stabilization of PINK1 at the surface of damaged mitochondria. In the years that followed, further light has been shed on the PINK1-dependent mitophagy mechanism, including the importance of PINK1-dependent phosphorylation of ubiquitin for mitochondrial Parkin recruitment and the subsequent activation of Parkin’s E3 ubiquitin ligase activity through direct phosphorylation by PINK1 [4].

**Fig 1:**
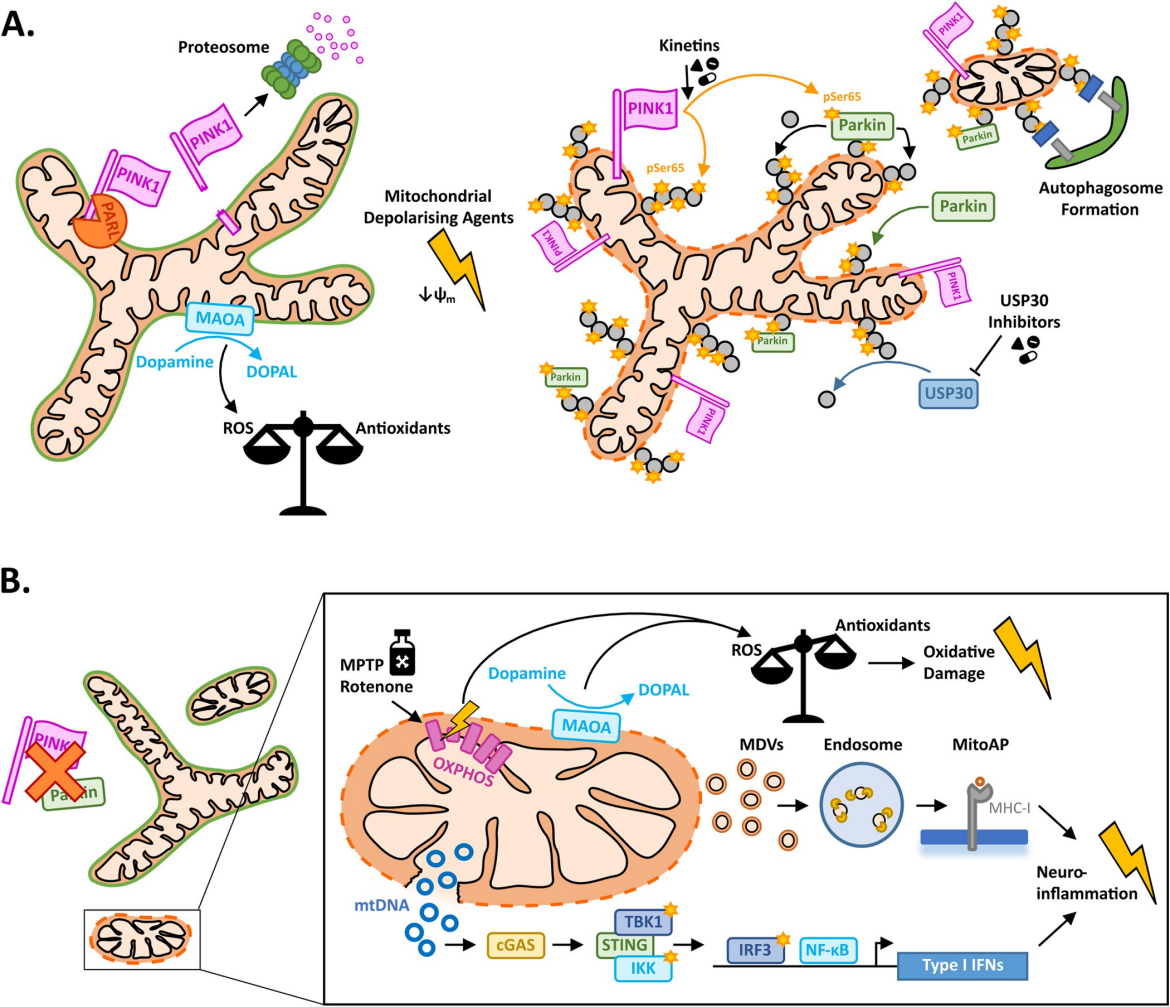
PINK1–Parkin-dependent mitophagy is crucial for dopaminergic neuron viability. (**A**) In healthy mitochondria with a polarized mitochondrial membrane potential (ψ_m_), PINK1 is cleaved by PARL on the inner mitochondrial membrane rendering it vulnerable to proteosome degradation. Upon mitochondrial membrane depolarization, PINK1 is instead stabilized at the outer mitochondrial membrane where it phosphorylates ubiquitin molecules (pUb(Ser65)). pUb(Ser65) serves as a recruitment site for Parkin, which is subsequently phosphorylated by PINK1 to activate it. Parkin deposits further ubiquitin chains at the mitochondria, which are also phosphorylated by PINK1, resulting in a positive feedback loop resulting in the coating of damaged mitochondria with pUb(Ser65). pUb(Ser65) serves as a signal for autophagosome formation, engulfment, and subsequent degradation of the damaged mitochondria through the autophagy–lysosome pathway. Kinetins serve as potent neosubstrates for PINK1 and can enhance PINK1-dependent activity, while inhibitors of the deubiquitinase USP30 can enhance mitochondrial ubiquitination. (**B**) Dopamine metabolism is associated with high levels of ROS production (e.g., mitochondrial MAOA catabolism of dopamine) rendering dopaminergic neurons particularly vulnerable to oxidative damage. Impairments in PINK1 or Parkin can result in the accumulation of damaged mitochondria, which release ROS and toxins that target OXPHOS complex I elevate mitochondria-derived ROS production. PINK1 and Parkin are also involved in attenuating release of MDVs for MitoAP and activation of adaptive immune responses. Finally, PINK1and Parkin prevent uncontrolled release of mtDNA by damaged mitochondria, which would otherwise activate the cGAS–STING pathway resulting in activation of type I IFN-mediated innate immune responses. IFN, interferon; MAOA, monoamine oxidase-A; MDV, mitochondria-derived vesicle; MitoAP, mitochondrial antigen presentation; ROS, reactive oxygen species.

Genetic analyses and subsequent functional studies have continued to strengthen the link between mitochondrial dysfunction and familial PD, with the products of other causative recessive and dominant genes also implicated in mitochondrial physiology [5]. Despite these observations, and the comparable clinicopathological phenotypes of individuals with idiopathic or familial PD, the relevance of mitophagy dysfunction in the context of idiopathic PD has remained somewhat unclear. Recent genome-wide association studies (GWAS) have greatly advanced our understanding surrounding the genetics of idiopathic PD risk [6]. These studies have revealed at least partial concordance of underlying disease mechanisms, with idiopathic PD risk loci tagging a number of genes that have well-established causative associations with familial PD, including *GBA1*, *SNCA*, and *LRRK2*. The *PINK1* and *PRKN* genomic loci are not tagged in idiopathic PD GWAS [6]; however, this does not refute the potential importance of mitophagy dysfunction in the context of idiopathic PD. Indeed, targeted functional screening for PINK1-dependent mitophagy initiation modifiers among PD GWAS candidate genes has recently identified KAT8 and KANSL1 of the nonspecific lethal epigenetic remodeling complex [7], and it will be interesting to understand whether genes at other idiopathic PD risk loci are also involved in the mitophagy process. These data not only support an involvement of PINK1–Parkin-dependent mitophagy dysfunction in idiopathic PD but also show that PD genetics still has more to give to further advance our mechanistic understanding of PINK1–Parkin-dependent mitophagy.

Despite these great advances, it is unclear how much PINK1 and Parkin contribute to mitophagy under normal physiological conditions in vivo, and what their precise function in dopaminergic neurons might be [8]. Much of our knowledge surrounding the PINK1–Parkin-dependent mitophagy process has come from in vitro studies utilizing cell lines that overexpress Parkin and which have been treated with high doses of mitochondrial toxins to completely collapse the membrane potential of the mitochondrial network. Under healthy basal conditions, PINK1–Parkin-dependent mitophagy is likely a rare event, with the dynamic nature of the mitochondrial network and concerted activity of fission–fusion and mitochondrial biogenesis processes sufficient to maintain the majority of mitochondrial units in a polarized state. However, this does not curtail the importance of PINK1 and Parkin for dopaminergic neuron survival.

The heavy oxidative requirement of dopamine metabolism, a process linked with the production of high levels of reactive oxygen species (ROS), predisposes dopaminergic neurons to oxidative damage and, consequentially, to neurodegeneration (Fig 1B). For example, mitochondrial monoamine oxidase A (MAO-A) has an important role in dopamine catabolism and is a major source of ROS. This could not only explain the vulnerability of dopaminergic neurons to mitochondrial dysfunction and/or accumulation of ROS producing damaged mitochondria in the context of PINK1–Parkin-dependent mitophagy impairments, but likely also accounts for why mitochondrial toxins targeting oxidative phosphorylation complex I that elevate ROS (e.g., paraquat, MPTP, rotenone) also lead to selective dopaminergic neurodegeneration. Aging represents the greatest risk factor for idiopathic PD, and while impairments in PINK1–Parkin-dependent mitophagy might be difficult to detect experimentally under basal conditions, cumulative oxidative cellular damage associated with long-term deficits in the removal of damaged mitochondrial components fits mechanistically with observations of age-related dopaminergic neurodegeneration in PD.

PINK1 and Parkin knockout mice do not recapitulate the dopaminergic neuron degenerative phenotypes of humans with PD, and in vivo mitophagy measures have proven to be largely unaffected in these mice [8]. However, when Parkin knockout animals are crossed onto a *Polg* mutant mutator background that accumulates dysfunctional mitochondria, dopaminergic neurodegeneration and parkinsonism-like motor phenotypes are observed [9]. Intriguingly, a key pathway associated with dopaminergic neurodegeneration in this model is the cGAS–STING arm of the innate immune response, which is activated by cytosolic DNA (such as that released from damaged mitochondria). Thus, impairments in PINK1 or Parkin and, presumably, the associated impairments in the controlled degradation of damaged mitochondria lead to consequential release of mitochondrial DNA into the cytoplasm. In line with this, elevated levels of circulating cell-free mitochondrial DNA have been observed in the serum of patients with PD harboring biallelic *PINK1* and *PRKN* mutations, and this subset of patients also shows particularly high levels of the pro-inflammatory cytokine IL-6 [10]. Interestingly PINK1 and Parkin have also been linked with regulating activation of adaptive immune responses by preventing the formation and release of small double membrane mitochondrial-derived vesicles that are trafficked into endosomes for mitochondrial antigen processing and subsequent cell surface presentation [11]. Chronic neuroinflammation is a frequent observation in the brain of patients with PD and, for a long while, was thought to be a secondary consequence arising due to immune system activation by degenerating neurons. These data might also highlight a primary function of immune system activation associated with PINK1–Parkin-dependent mitophagy impairments in PD pathogenesis.

While the precise consequences of the PINK1 and Parkin impairments that are most important in relation to dopaminergic neurodegeneration still remain to be determined, enhancement of PINK1 and Parkin activity is an attractive avenue for novel disease-modifying therapies in PD. Several different targeted strategies are undergoing preclinical investigation, including allosteric enhancers of Parkin, inhibitors of the mitochondrial targeted deubiquitinase USP30, enhancement of PINK1 activity with kinetin based substrates, and stabilization of PINK1 with inhibitors of FBXO7 [5]. Other strategies aiming to enhance mitophagy in a PINK1–Parkin-independent manner are also being explored; however, the described cellular specificity of PINK1–Parkin-dependent activity at damaged mitochondria, and particularly that of PINK1 as the upstream mitochondrial damage sensor [3], could make these targets the most efficacious and safest routes for clinical intervention in PD.

Early work, including that of Narendra and colleagues [3], laid the foundations of PINK1-dependent mitophagy research. Our understanding of PD pathomechanisms and the complexities of the mitophagy process continue to improve in parallel with one another and only strengthen the already well-established causative link between mitophagy dysfunction and PD.

## Abbreviations

GWAS: genome-wide association studies
MAO-A: monoamine oxidase A
PD: Parkinson’s disease
ROS: reactive oxygen species
